# Influence of Tool Holder Types on Vibration in Rough Milling of AZ91D Magnesium Alloy

**DOI:** 10.3390/ma14102517

**Published:** 2021-05-12

**Authors:** Ireneusz Zagórski, Jarosław Korpysa, Andrzej Weremczuk

**Affiliations:** 1 Department of Production Engineering, Mechanical Engineering Faculty, Lublin University of Technology, 36 Nadbystrzycka Str., 20-618 Lublin, Poland; j.korpysa@pollub.pl; 2 Department of Applied Mechanics, Mechanical Engineering Faculty, Lublin University of Technology, 20-618 Lublin, Poland; a.weremczuk@pollub.pl

**Keywords:** magnesium alloys, rough dry milling, vibrations, composite multiscale entropy, tool holders

## Abstract

The article presents the results of an analysis of the influence of the technological parameters related to tool holder types on the vibrations occurring during the milling of AZ91D magnesium alloy. Magnesium alloys are very low-density materials and, therefore, are increasingly being considered as replacement materials for the more commonly used aluminium alloys. The tool used in the study was a carbide end mill with TiAlN coating, clamped in three different types of tool holder: ER collet, heat shrink, and Tendo E hydraulic. The milling tests used straight toolpaths at varied cutting speeds and feed per tooth values. Based on the vibration displacement and acceleration signals recorded during the machining tests, the following were analysed: maximum value, amplitude, and root mean square (RMS) value of the vibrations. As part of the study, composite multiscale entropy (CMSE) analysis was also performed, describing the level of disorderliness of the obtained vibration signals. The increase in machining parameters caused an increase in the values characterising the displacement and acceleration of the vibrations. It was noted that multiscale entropy might be an important parameter describing the vibration signal (both displacement and acceleration).

## 1. Introduction—State of the Art

Manufacturers of modern machines and devices are constantly facing new challenges. There is continuous striving for improvement and the introduction of new, state-of-the-art, and innovative designs, which are also characterised by light structures where possible. Light construction facilitates a reduction in fuel consumption for the automotive, aviation, and space industries. In the course of manufacturing such elements or entire structures, undesirable phenomena may occur in the form of self-excited vibrations generated during the machining processes (including the most unfavourable form: chatter).

The stability of the milling process can be affected by multiple factors, including machining strategy, machine tool type, machining fixture, tool and fitting, workpiece, cooling, and lubrication of the tool-workpiece interface and production parameters [1,2]. Stability analyses are also often performed for thin-walled elements. Such tests are often carried out for various grades of aluminium alloy, including EN AW-7075 T6/T7 [3,4,5], EN AW-2024-T351 [6], and EN AW-6061-T6 [7,8]. For example, in one study [3], the stability limit was determined for elements made of Al-7075 alloy featuring decreased wall thicknesses. Moreover, quantitative data analyses from recurrence quantification analysis (RQA) and recursive plots were presented. In the case of machining thin-walled elements, the change in the modal parameters might be of crucial importance for stability lobe diagram prediction.

In the world literature, there is much discussion on the analysis of vibrations during the machining processes of difficult-to-machine materials, such as titanium alloys, nickel alloys, and high chromium steel. Chatter-type vibrations can effectively prevent the processing of light alloys, including aviation aluminium and magnesium alloys. In general, self-excited chatter vibrations are considered to be one of the main obstacles in the machining process, significantly affecting the course and efficiency of machining. In extreme cases, it may lead to a loss of control over the machining process. As a consequence, it is not possible to predict the properties of the finished product, such as dimensional and shape accuracy as well as surface quality [1,9]. Moreover, this situation can be of particular importance in the case of precision and ultraprecision machining of low-rigidity elements. The main reasons for the loss of stability in the course of machining include excessive friction (at the tool-workpiece interface), displacement feedback (vibration in the direction of the passive force F_p_ generating vibrations in the direction of the main cutting force F_c_ and vice versa) and the phenomenon of trace regeneration (due to vibrations causing a variable thickness of the machined layer) [10,11]. Chatter-type vibrations can lead to the occurrence of characteristic notches on the machined surface, mainly as a result of vibrations arising between the tool and the workpiece. Therefore, in addition to the deterioration of the machined surface quality, vibration can affect the durability and service life of the machine components, accelerate their wear, as well as cause faster or even catastrophic tool wear (mainly of the surfaces actively involved in the machining process). Vibration amplitudes that are too high may also result in damage to the tool holders and machining units, as well as posing a threat to the health of the machine operators [12]. Another harmful factor is excessive noise, especially at high frequencies [13]. The occurrence of vibration is often associated with the necessity to limit the applied technological parameters, significantly reducing the efficiency of the process, hence extending the machining time and reducing the efficiency and effectiveness of the machining process [14].

It is commonly believed that the methods of eliminating self-excited vibration can be divided into two basic groups. The first group comprises methods based on the selection of an appropriate configuration for the technological parameters to ensure a stable process. From these can be distinguished a subgroup of techniques used outside the cutting process, including stability forecasting based on stability charts, process modelling, or analytical methods. The second subcategory covers intra-process techniques, comprising the ongoing correction of machining parameters, e.g., on the basis of signals measured by sensors. The second group includes methods of introducing various types of change to the system and shifting the stability limit. These include active methods—based on the continuous modification of system behaviour, such as by changing the rotational speed or feed rate, and passive methods, such as requiring changes to the design of the tools, holders, or machines as well as the use of additional equipment. The vibration elimination methods can also be classified, where group one includes methods ensuring the stability of the machining process (parameter selection, stability lobe diagram—SLD) as well as those that use the lobbying effect; group two covers all methods related to changing system behaviour, as well as modifying the stability limit [10]. Various types and methods of chatter vibration damping are included in [15]. The latter also includes a description of the manner of selecting the vibration damping method, depending on various conditions occurring as part of the machining process (workpiece machinability and machining system rigidity). Lobe curves (SLD) can also take into account the influence of the cutting tool (milling cutter) diameter. It was observed that an increase in diameter may increase the area corresponding to stable machining, while an increase in the number of tool teeth increases significantly the useful range of the rotational speed n (SLD curves shift to the right) [16]. Unfortunately, there are also some disadvantages associated with the use of SLD. These include the static approach to obtaining the input data for determining the SLD curves, i.e., at machine standstill (static test). Therefore, the key dynamic parameters of the spindle system (rigidity, thermal expansion) and various factors related to the workpiece (damping properties, material defects, and changes in the cross-section of the machined layer) are not taken into account [15,17]. Therefore, it would seem particularly important to verify a theoretical lobe curve during a dynamic cutting test under machine tool real-life conditions. The methods presented in [18], i.e., the chase plane method, Poincare method, and spectra analysis are often used in studies on the stability of the milling process. That article discussed the correlation between the maximum Lyapunov exponent and the milling parameters (spindle speed and milling depth). The domain of vibration stability in the milling process was determined using the criterion of the greatest Lyapunov exponent (a value of 0.61 indicated a non-linear vibration criterion). The exponent value grew with an increase in a_p_. For example, external enforcement could be used to control the milling process [11]. Numerical studies were carried out in Matlab-Simulink (fourth-order Runge-Kutta method with a variable integration step for a non-linear model with two degrees of freedom).

Nonlinear phenomena, as well as tool and workpiece susceptibility, were taken into account. Stability diagrams were created, and the influence of the external enforcement was analysed. Proportional-derivative (PD) control (for closed-loop control) was used. A decrease in the vibration level during machining was observed by using a combination of external enforcement, correctly selected technological parameters (based on lobe curves), and the use of a PD controller [1].

**Figure 1:**
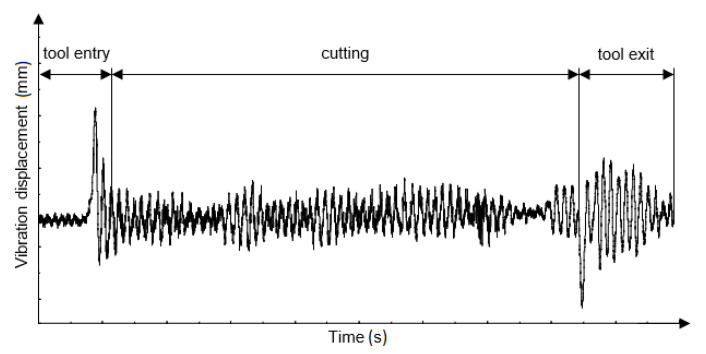
The waveform of the vibration displacement signal presented as a function of time.

The vibration signal is frequently used as a diagnostic signal and can be successfully used to replace the equally popular measurement of total machining force components. An additional advantage of the vibration signal is that measuring the machining force components usually requires the use of quite expensive force gauges, which are also difficult to install. The vibration signal method offers a good correlation with the force signal as well as the possibility of obtaining other information about the course of the process [19,20]. The measurement of the vibration amplitude or the root mean square value provides valuable information about the course of the process and correlates well with the force signal; however, it is more susceptible to disturbances (due to the sensor location) [1]. Generally, tactile sensors (e.g., accelerometers) or laser sensors (e.g., laser vibrometers) are used typically to carry out vibration measurements. The signals captured by the sensors can be analysed as a function of time or frequency [21]. An example vibration displacement signal waveform in the time domain is shown in Figure 1.

Entropy can also serve as a useful tool in the research and analysis of dynamic processes. The currently used method is multiscale entropy (MSE), defined as a measure of the degree of disorder or uncertainty of a given signal. It enables the assessment of the complexity of a time series and better understanding of the phenomena taking place. The multiscale entropy analysis is based on the coarse-graining procedure, which involves the averaging of the original points in a time series. Despite the successful use of this method in many areas, the measurements carried out with this method may be flawed due to a too high τ-scale factor. The solution to this problem is the development of a concept known as composite multiscale entropy (CMSE) which, unlike MSE, takes into account all coarse-grained series instead of only the first one. This method provides greater accuracy for larger scale factors [18,22].

Magnesium alloys are often used in the automotive and aerospace industries (e.g., [1], gear housing of steering gear, clutches, gears, and components of combustion engines, the cockpit control panel, or transmission). They are also an interesting and innovative construction material. The temperature in the cutting zone is a critical factor in the machinability of magnesium alloys. Nevertheless, such machinability indicators as cutting force components or vibrations are indirectly related to the generated temperature in the cutting zone. They can also be used to assess the stability of the milling process and thus to identify safety when machining magnesium alloys. The implementation of research on magnesium alloys made it possible to extend the current knowledge of their processing and thus increased the interest in these materials, which may result in their use on a wider scale. In addition, magnesium alloys belong to the modern, innovative materials of a new generation; hence they are an interesting research and construction material. This is mainly due to their low density, so that magnesium alloy components can successfully replace those made of aluminium alloys, mainly in applications where weight reduction is a key factor. Furthermore, they are characterised by very good machinability, corrosion resistance, and the ability to damp vibrations so they can be used in various industries [2].

Thus, on the basis of the literature review, some shortcomings could be identified in the analysis of the machinability of light alloys, including magnesium alloys, using vibration signal analysis, and composite multiscale entropy analysis (CMSE). A significant number of publications focused on other light metal group materials—aluminium alloys, and the analysis concerned only one parameter characterising the vibrations, usually the amplitude. This approach is insufficient for a detailed description of the vibrations that occur during the machining process. The number of works attempting to determine the effect of the type of tool mounting on the occurrence of vibrations was also very limited. Additionally, no publication was found regarding CMSE analysis in this regard. This suggested the need to broaden the available knowledge on the stability of the milling process in relation to magnesium alloys. The main aim of the article and research was vibration analysis, taking into consideration basic and additional indicators describing vibrations during machining. Moreover, an additional aim was to analyse the impact of changing technological parameters and the type of tool holder on the selected vibration indicators. In Section 1, a literature review was conducted. Section 2 contained technical details of the measurements and tests performed. The Results and Section 3 presented the main test results and the analysis of the obtained vibration results (the main chapter was divided into subsections: Vibration displacement, Vibration acceleration, Composite multiscale entropy). Section 4 contained the main conclusions in the form of main points.

## 2. Materials and Methods

The study used AZ91D (MgAl_9_Zn_1_) magnesium alloy. The dimensions of the test specimens were 112 mm × 150 mm × 56 mm. Milling operations were performed on an AVIA VMC 800 HS vertical machining centre (Warsaw, Poland) with Heidenhain iTNC 530 control (Dr. Johannes Heidenhain GmbH, Traunreut, Germany) (power 25 kW, n_max_ up to 24,000 rpm, and v_f max_ up to 40 m/min). Three types of tool holders with a HSK-A63 spindle were used for tool clamping: ER 32 × 100 SECO collet chuck (Fagersta, Sweden), SFD 16 × 120 SECO heat shrink chuck (Fagersta, Sweden), and Tendo E compact 20 × 80 Schunk hydraulic chuck (Lauffen/Neckar, Germany). All the tool holders were normally balanced by the manufacturers as G2.5 class for rotational speeds of 25,000 rpm in accordance with the ISO 21940-11: 2016 [23] standard. Prior to machining, the degree of unbalance was checked for all tool holder combinations using the CIMAT RT 610 balancing machine. The imbalance value for the tool mounted in the holder with the ER collet was 7.67 gmm, heat shrink SFD chuck was 4.27 gmm, and Tendo E hydraulic chuck was 7.24 gmm, corresponding to class G6.3 for a rotational speed of 20,000 rpm. Table 1 shows the chemical composition of the machined AZ91D magnesium alloy.

With respect to cutting data, the range of changeable milling parameters included: cutting speed v_c_ = 400–1000 m/min, feed per tooth f_z_ = 0.05–0.30 mm/tooth, and axial depth of the cut a_p_ = 6 mm. The selection of technological parameters was dictated partly by the rotational speed of the machining centre spindle, as well as the analysis of the generated lobe curves. The radial depth of the cut was constant at a_e_ = 14 mm. For change v_c_, the constant f_z_ was 0.15 mm/tooth, and for change f_z_, the constant v_c_ was 800 m/min. Milling operations were carried out with a straight shank solid carbide (VHM) end mill by Fenes (Siedlce, Poland) with TiAlN coating, recommended for aluminium and magnesium milling applications. It was a 2-flute tool, diameter d = 16 mm, overall dimensions 16 × 25 × 100 mm^3^, and helix angle λ_s_ = 30°. The milling process was performed as down-milling, along with straight toolpaths.

**Figure 2:**
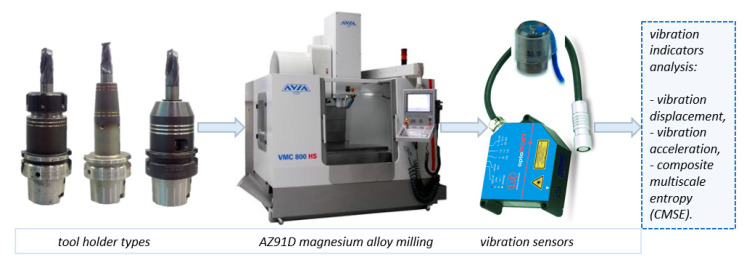
Schematic diagram of the test set-up.

Figure 2 shows the schematic diagram of the test system and the test set-up. The measurement apparatus used in the study was: for vibration acceleration measurement—PCB 352B10 accelerometer by Piezotronics (New York, NY, USA), for vibration displacement—optoNCDT LD1605-2 laser sensor by Micro-Epsilon Masstechnik (Ortenburg, Germany).

For the tests, vibration displacement and acceleration measurements were performed on the Y-axis (along the machine tool axis). The basic specifications of the PCB 352B10 sensor were [1]: sensitivity 1.02 mV/(m/s^2^), measurement range ±4905 m/s^2^ pk, frequency range 2–10,000 Hz, resonant frequency ≥65 kHz, and broadband resolution 0.03 m/s^2^ rms. The specifications of the laser-optical sensor, optoNCDT LD1605-2, were measurement range 2 mm, resolution from 0.5 μm, frequency response and the sampling rate 10 kHz, and spot diameter 0.3 mm. Modal analysis is a process typically employed in various research works with the purpose of investigating the basic dynamic parameters of given systems as well as generating stability lobe diagrams. The SLD analysis was performed with the use of CutPro software (Manufacturing Automation Laboratories Inc., Vancouver, Canada), a 352B10 PCB accelerometer (PCB Piezotronocs, Inc., New York, NY, USA), and a 070A02 PCB scope input adaptor (PCB Piezotronocs, Inc., New York, NY, USA).

In the time series obtained during the measurements, the cutting areas of the fixed machining allowance were examined, excluding the moments of tool entry and exit from the workpiece. The following parameters of vibration displacement and acceleration were analysed: maximum value of displacement, acceleration x_y_/a_y_, the amplitude of displacement/acceleration A_xy_/A_ay_, and root mean square value of displacement/acceleration x_y_RMS_/a_y_RMS_.

During the analysis of vibration displacement and acceleration, the amplitude was determined as half the difference between the maximum and minimum value, according to Equations (1)–(8):

(1) $${\mathrm{Ax}}_{y}=\frac{\left(x_{y\_\max}-x_{y\_\min}\right)}{2}$$

(2) $${\mathrm{Aa}}_{y}=\frac{\left(a_{y\_\max}-a_{y\_\min}\right)}{2}$$

where: ${\mathrm{Ax}}_{y}$—displacement amplitude, ${\mathrm{Aa}}_{y}$—acceleration amplitude, $x_{y\_\max}$—maximum displacement value, $a_{y\_\max}$—maximum acceleration value, $x_{y\_\min}$—minimum displacement value, $a_{y\_\min}$—minimum acceleration value.

The root mean square value of the vibration displacement and acceleration was determined from the following correlation:

(3) $$x_{y\_\mathrm{RMS}}=\sqrt{\frac{1}{T}\int_{0}^{T}x_{y}^{2}\left(t\right)\mathrm{dt}}$$

(4) $$a_{y\_\mathrm{RMS}}=\sqrt{\frac{1}{T}\int_{0}^{T}a_{y}^{2}\left(t\right)\mathrm{dt}}$$

where: $x_{y_{\mathrm{RMS}}}$—root mean square value, $a_{y_{\mathrm{RMS}}}$—rms acceleration value, x(t)—displacement value as a function of time t, a(t)—acceleration value as a function of time t.

The CMSE was determined taking into account all coarse-grained series according to the following correlation:

(5) yk, j(τ)=1τ∑i=(j−1)τ+ki=jτ+k−1xi, 1≤j≤Nτ, 1≤k≤τ

where: τ—scale factor, N—length of a data series, and x is a raw one-dimensional time series x *=* {x_1_, x_2_, …, x_N_}.

Then the composite multiscale entropy (CMSE) took the following form:

(6) $$\mathrm{CMSE}\left(x,\tau ,m,r\right)=\frac{1}{\tau }\overset{\tau }{\underset{k=1}{\sum }}\mathrm{SampEn}\left(y_{k}^{\left(\tau \right)},m,r\right)$$

where: m—pattern length, r—similarity criterion.

Calculation of the N_d_ and N_n_ values from the previously prepared coarse-grained data y^(τ)^ was carried out according to the following procedure:

(7) Nd=Nn=1   if |y(τ)(i)−y(τ)(j)|<r ^ |y(τ)(i+1)−y(τ)(j+1)|<r  Nn=Nn+1;if |y(τ)(i+2)−y(τ)(j+2)|<r  Nd=Nd+1;

The sampling entropy (SampEn) in Equation (6) was defined according to the following equation:

(8) $$\mathrm{SampEn}\left(y_{k}^{\left(\tau \right)},m,r\right)=\ln\left(\;\frac{N_{n}}{N_{d}}\right)$$

**Figure 3:**
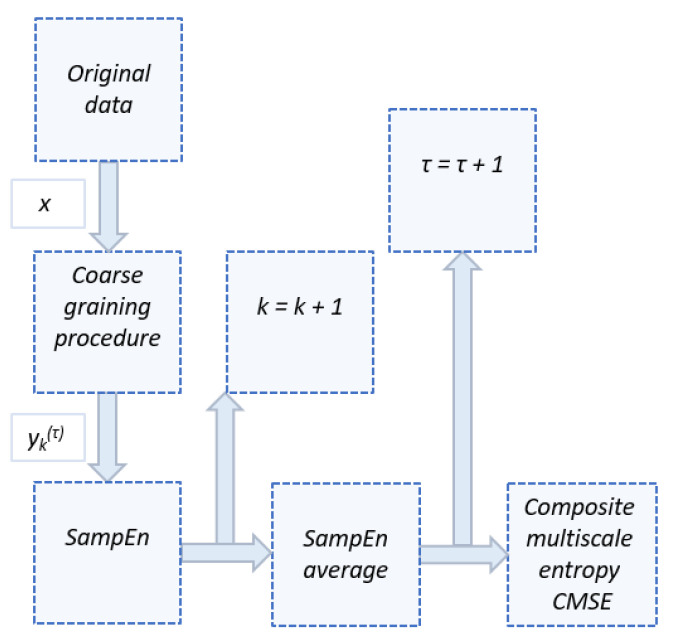
Composite multiscale entropy (CMSE) calculation algorithm.

The algorithm for calculating the composite multiscale entropy (CMSE) is presented in the form of the block diagram in Figure 3, where the final result for each scale factor *τ* is the averaged SampEn depending on k-th loops.

The MATLAB software was used to perform the CMSE calculations. During the analysis, the length of the chains was m = 2, the scale factor τ = 50, and the similarity tolerance r = 0.1 σ_x_, where σ_x_ was the standard deviation of the original time series [18,21,24].

## 3. Results and Discussion

**Figure 4:**
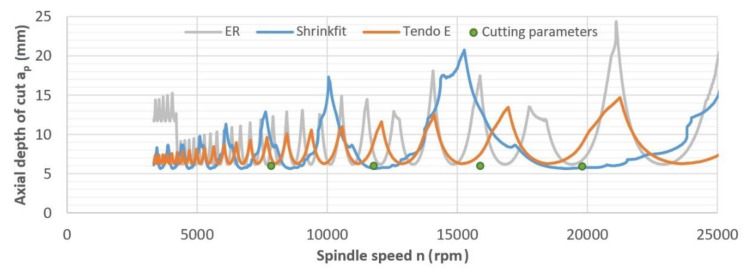
Stability curves for the tool mounted in ER collet chuck, heat shrink chuck, and Tendo E hydraulic chuck (f_z_ = 0.15 mm/tooth).

Figure 4 shows SLD diagrams for the cutting tool parameters and each type of tool holder. The markings in the chart defined the given machining regions as stable—in the upper part of the chart above the stability lobes (often marked as +), unstable—below the stability lobes (often marked as −), and the settings selected as milling parameters. Operating within these established parameters, i.e., in this case, rotational speed n and axial depth of cut a_p_ should ensure that the system remained stable throughout the milling process. However, the SLD analysis is carried out during static tests and fails to account for the dynamics of the machining system (resulting from the complexity of the machine tool and of the milling process itself), thus it was especially important to perform a real milling test under specified machining conditions.

**Figure 5:**
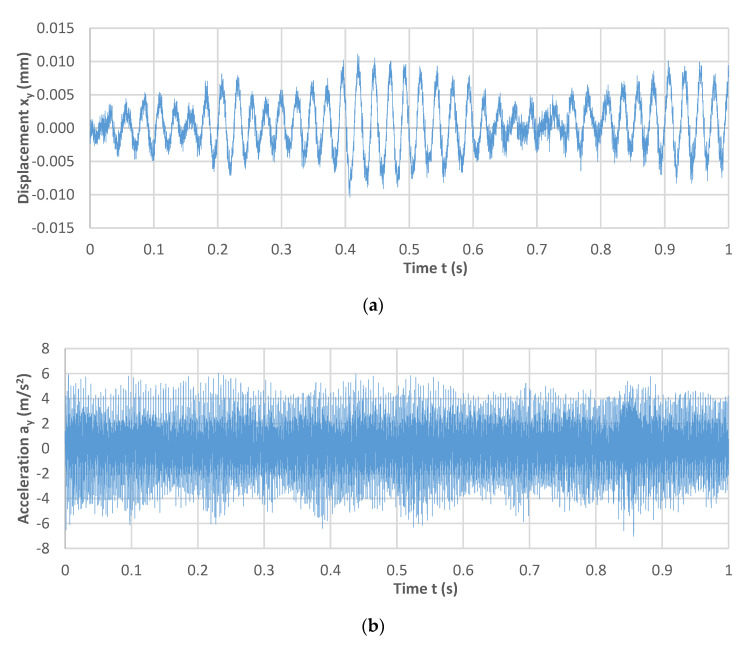
Examples of time series for (**a**) displacement, (**b**) acceleration of vibrations recorded during the milling process (v_c_ = 800 m/min, f_z_ = 0.05 mm/tooth, a_p_ = 6 mm).

Figure 5 shows examples of vibration displacement and acceleration time series recorded during milling with a cutting speed v_c_ = 800 m/min, feed per tooth f_z_ = 0.05 mm/tooth, and axial depth of cut a_p_ = 6 mm.

While registering the vibration displacement, a modulated signal was obtained; while in the case of vibration acceleration, the registered signal did not show any sudden changes in its values, with the average value remaining approximately constant.

### 3.1. Vibration Displacement

**Figure 6:**
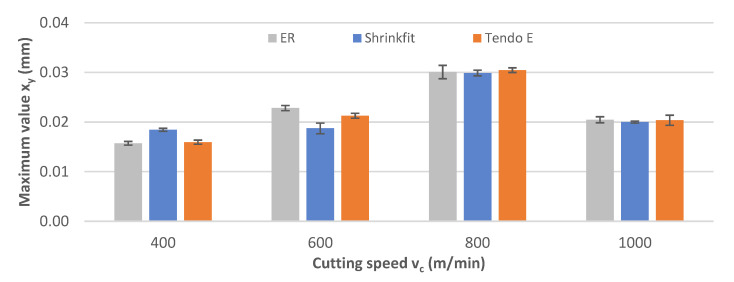
Impact of cutting speed v_c_ on maximum vibration displacement value x_y_ (f_z_ = 0.15 mm/tooth, a_p_ = 6 mm).

The impact of cutting speed on the vibration displacement was tested over the range v_c_ = 400–1000 m/min. Figure 6 shows the maximum vibration displacement values x_y_ obtained while milling using each of the three tool holders.

Similar values were obtained for all three tool holders in the analysis of the maximum vibration displacement values. The lowest values were recorded for the heat shrink chuck while milling at the speeds of v_c_ = 600 m/min and v_c_ = 1000 m/min, and the collet chuck at the speeds of v_c_ = 400 m/min, and v_c_ = 800 m/min. By increasing the cutting speed, the maximum values also increased steadily, with a slight decrease at the highest speed of v_c_ = 1000 m/min.

**Figure 7:**
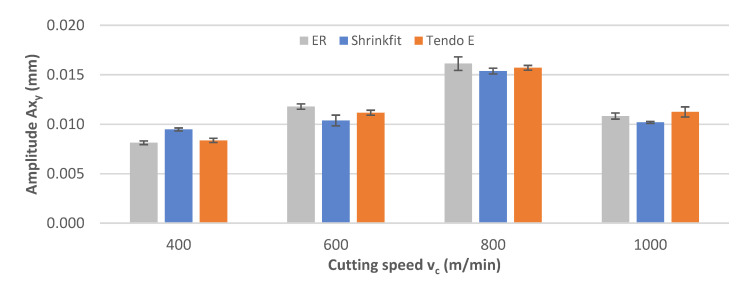
Impact of cutting speed v_c_ on the vibration displacement amplitude Ax_y_ (f_z_ = 0.15 mm/tooth, a_p_ = 6 mm).

Figure 7 shows the impact of the cutting speed change on the vibration displacement amplitude Ax_y_ during the milling process. While milling at a speed of v_c_ = 400 m/min, the lowest amplitude values were recorded for the ER collet chuck and for the heat shrink chuck in the case of other speeds. Throughout the range of cutting speed changes, similar values were obtained for all tool holders, increasing with higher cutting speed values, except for at the highest speed, which was associated with a slight decrease.

**Figure 8:**
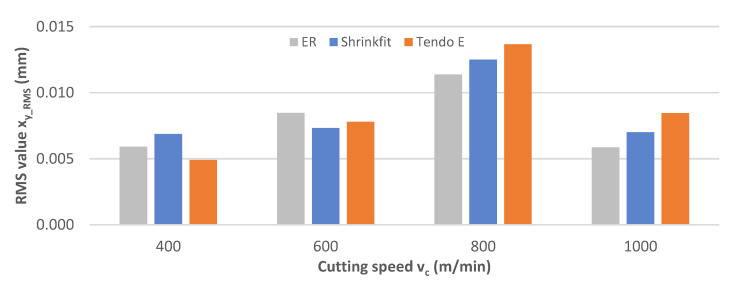
Impact of cutting speed v_c_ on rms vibration displacement value x_y_RMS_ (f_z_ = 0.15 mm/tooth, a_p_ = 6 mm).

The impact of the cutting speed change on rms displacement values x_y_RMS_ is shown in Figure 8. The cutting speed increase resulted in a gradual value increase up to the speed of v_c_ = 800 m/min, followed by a slight decrease. The achieved rms values were 0.005–0.014 mm, with the lowest values for the hydraulic chuck while machining at a speed of v_c_ = 400 m/min, the heat shrink chuck at v_c_ = 600 m/min, and the ER collet chuck at v_c_ = 800–1000 m/min.

**Figure 9:**
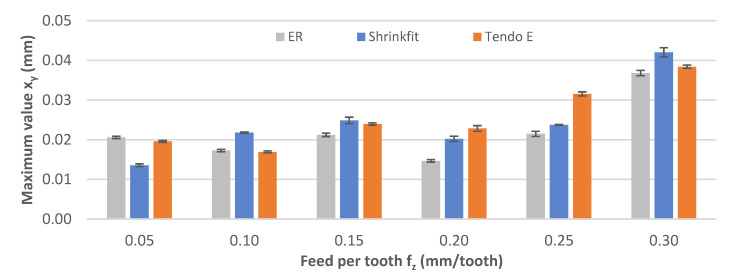
Impact of feed per tooth f_z_ on maximum vibration displacement value x_y_ (v_c_ = 800 m/min, a_p_ = 6 mm).

The impact of the feed per tooth on vibration displacement was tested over the range f_z_ = 0.05–0.30 mm/tooth. The obtained maximum vibration displacement values x_y_ are shown in Figure 9.

Increasing the feed per tooth to f_z_ = 0.10 mm/tooth resulted in a vibration displacement value decrease, while further ranges were associated with a continuous increase. While carrying out the milling process, the recorded values were 0.014–0.042 mm, with the lowest feed per tooth values of f_z_ = 0.15–0.30 mm/tooth for the ER collet chuck, f_z_ = 0.05 mm/tooth for the heat shrink chuck, and f_z_ = 0.10 mm/tooth for the Tendo E hydraulic chuck.

**Figure 10:**
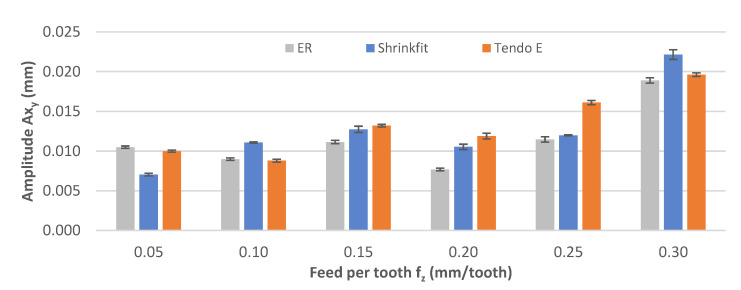
Impact of feed per tooth f_z_ on the vibration displacement amplitude Ax_y_ (v_c_ = 800 m/min, a_p_ = 6 mm).

Figure 10 shows the impact of the feed per tooth change on the vibration displacement amplitude Ax_y_. In the course of machining, the recorded amplitude values were 0.007–0.022 mm, with the lowest values recorded mostly for the ER collet chuck at f_z_ = 0.15–0.30 mm/tooth. Increasing the feed per tooth resulted in an initial reduction in the value (f_z_ = 0.10 mm/tooth) followed by an increase in amplitude; however, a decrease also occurred for the tooth feed per tooth value of f_z_ = 0.20 mm/tooth.

**Figure 11:**
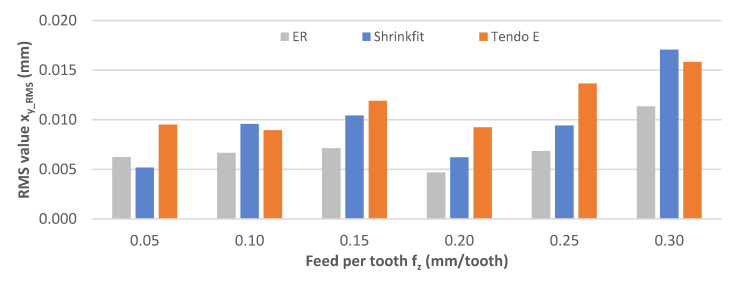
Impact of feed per tooth f_z_ on the rms vibration displacement value x_y_RMS_ (v_c_ = 800 m/min, a_p_ = 6 mm).

The rms vibration displacement values x_y_RMS_ obtained at a variable feed per tooth are shown in Figure 11. During the machining process, the recorded values were 0.012–0.023 mm, with the lowest feed per tooth of f_z_ = 0.05–0.15 mm/tooth for the heat shrink chuck, f_z_ = 0.20 mm/tooth for the hydraulic chuck, and f_z_ = 0.25–0.30 mm/tooth for the ER collet chuck. Increasing the feed per tooth resulted in a gradual increase in the maximum values, with the exception of f_z_ = 0.20 mm/tooth, which demonstrated a decrease.

### 3.2. Vibration Acceleration

**Figure 12:**
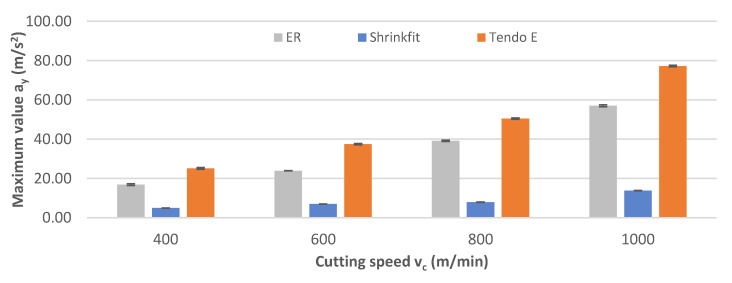
Impact of cutting speed v_c_ on the maximum vibration acceleration value a_y_ (f_z_ = 0.15 mm/tooth, a_p_ = 6 mm).

The impact of cutting speed on maximum vibration acceleration values a_y_ obtained while milling with the use of a tool mounted in the three tool holders is shown in Figure 12.

Increasing the cutting speed resulted in an increase in the recorded maximum vibration acceleration values. The lowest values of 4.90–13.76 m/s^2^ were measured while milling with the heat shrink chuck, while the highest values of 25.15–77.22 m/s_2_ were measured for the Tendo E hydraulic chuck.

**Figure 13:**
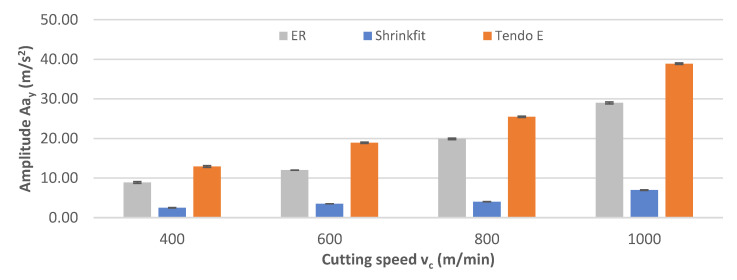
Impact of cutting speed v_c_ on the vibration acceleration amplitude Aa_y_ (f_z_ = 0.15 mm/tooth, a_p_ = 6 mm).

Figure 13 shows the impact of the cutting speed change on vibration acceleration amplitudes Aa_y_ during the milling process. The change in cutting speed resulted in a pronounced increase in the amplitude, which mainly applied to the ER collet and the hydraulic chucks, with the values of 11.30–26.35 m/s^2^, while for the heat shrink chuck, the amplitude was 2.30–6.97 m/s^2^.

**Figure 14:**
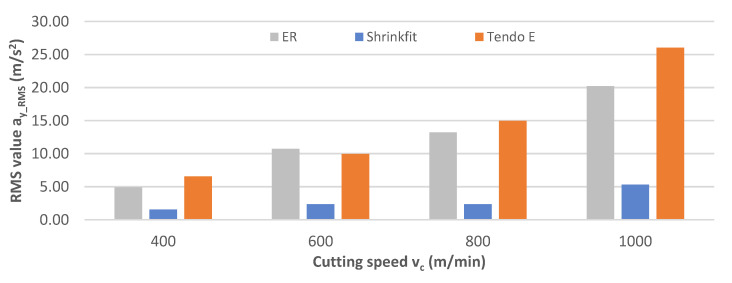
Impact of cutting speed v_c_ on the rms vibration acceleration value a_y_RMS_ (f_z_ = 0.15 mm/tooth, a_p_ = 6 mm).

Figure 14 shows the obtained rms vibration acceleration values a_y_RMS_ while milling at a variable cutting speed. While milling with the ER collet and Tendo E hydraulic chucks, the values increased with increases in the cutting speed much faster than in the case with the heat shrink chuck, for which the values were several times lower, at 1.57–5.34 m/s^2^.

**Figure 15:**
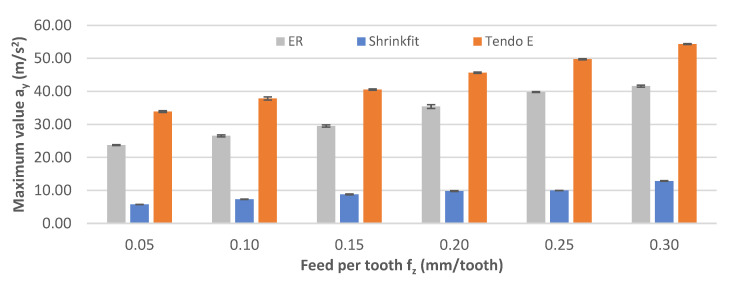
Impact of feed per tooth f_z_ on the maximum vibration acceleration value a_y_ (v_c_ = 800 m/min, a_p_ = 6 mm).

Figure 15 shows the impact of feed per tooth on the maximum vibration acceleration values a_y_. Similarly to the cutting speed change, a linear value increase was observed as a result of feed per tooth increase. The lowest values of 5.73–54.33 m/s^2^ were recorded for the heat shrink chuck, while for the other chucks, the values were several times higher at 23.72–41.58 m/s^2^ for the ER chuck and 33.88–54.33 m/s^2^ for the Tendo E chuck.

**Figure 16:**
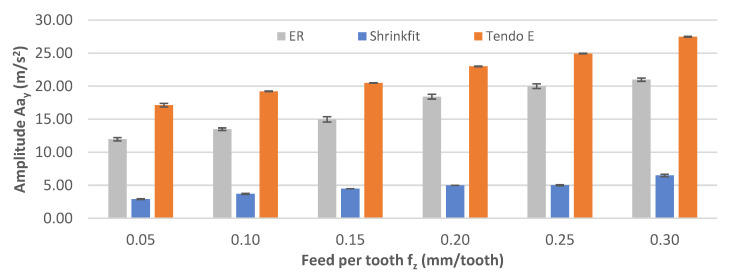
Impact of feed per tooth f_z_ on the vibration acceleration amplitude Aa_y_ (v_c_ = 800 m/min, a_p_ = 6 mm).

The impact of the feed per tooth change on vibration acceleration amplitude Aa_y_ is shown in Figure 16. The reported values increased over the entire range of the feed per tooth change, regardless of the tool holder used. The amplitude values were in the range 2.89–6.48 m/s^2^, 11.97–20.97 m/s^2^, and 17.12–27.48 m/s^2^ for the heat shrink, ER, and hydraulic holders, respectively.

**Figure 17:**
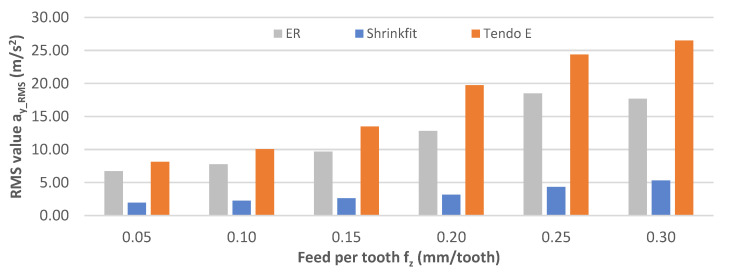
Impact of feed per tooth f_z_ on rms vibration acceleration value a_y_RMS_ (v_c_ = 800 m/min, a_p_ = 6 mm).

Figure 17 shows the impact of the feed per tooth on the rms vibration acceleration values a_y_RMS_. Over the entire range of feed per tooth changes, the lowest values were obtained with the use of the heat shrink chuck, at 1.94–5.31 m/s^2^. While using the hydraulic and the ER chucks, the measured values were several times higher at 6.72–26.50 m/s^2^, with linear increases as a result of feed per tooth increases.

### 3.3. Composite Multiscale Entropy (CMSE)

Composite multiscale entropy (CMSE) was also used to analyse the vibration displacement and acceleration signals obtained during the milling tests as an alternative method of testing the stability of the machining processes. It is a measure of the level of disorder and complexity of the signals. CMSE was determined for a scale factor of τ = 50, but the analysis was carried out in the range of τ = 1–20 due to the most significant changes occurring in this area.

**Figure 18:**
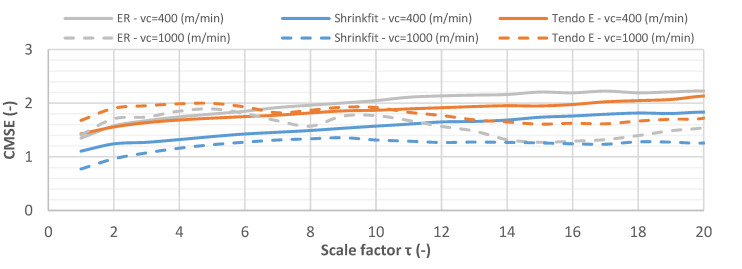
CMSE for vibration displacement signals while milling at v_c_ = 400 m/min and v_c_ = 1000 m/min (f_z_ = 0.15 mm/tooth, a_p_ = 6 mm).

The change in CMSE for vibration displacement signals for milling at cutting speeds of v_c_ = 400 m/min and v_c_ = 1000 m/min is shown in Figure 18.

While milling at a cutting speed of v_c_ = 400 m/min, the entropy values for all vibration displacement signals increased gradually along with the increasing scale factor. Using v_c_ = 1000 m/min, the entropy values for the respective holders were initially higher but gradually decreased, with the exception of the heat shrink holder, which was characterised by the lowest level of disorder across the entire scale range.

**Figure 19:**
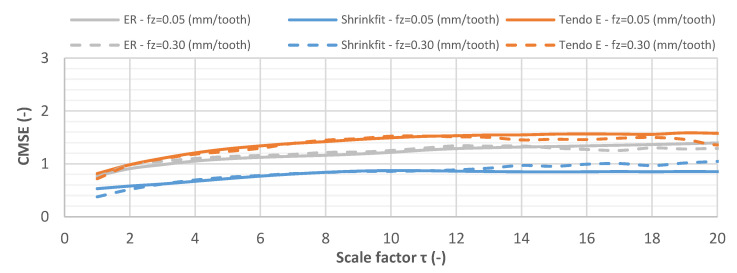
CMSE for vibration displacement signals while milling at f_z_ = 0.05 mm/tooth and f_z_ = 0.30 mm/tooth (v_c_ = 800 m/min, a_p_ = 6 mm).

Figure 19 shows the CMSE curve for the vibration displacement signals while milling at a feed rate of f_z_ = 0.05 mm/tooth and f_z_ = 0.30 mm/tooth. Initially, the entropy value increased for all vibration displacement signals, and then it stabilized at a higher scale factor. The signals recorded during the machining showed varied levels of regularity for the respective tool holders and were similar for both cutting speeds; however, the least disordered signal occurred during the machining with the milling cutter mounted in the heat shrink holder.

**Figure 20:**
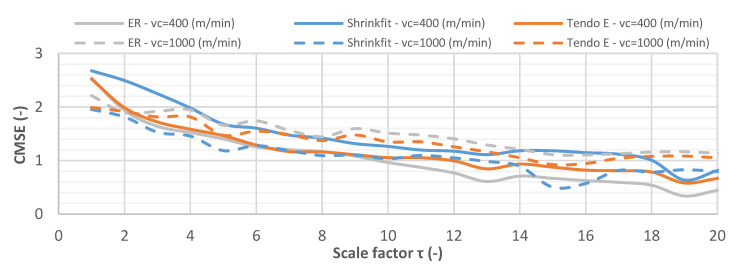
CMSE for vibration acceleration signals while milling at v_c_ = 400 m/min and v_c_ = 1000 m/min (f_z_ = 0.15 mm/tooth, a_p_ = 6 mm).

Figure 20 shows the CMSE curve for the vibration acceleration signals while milling at cutting speeds of v_c_ = 400 m/min and v_c_ = 1000 m/min. Contrary to the vibration displacement signals, the entropy value decreased gradually for all vibration acceleration signals, which indicated a decrease in the level of their disorder. The recorded signals showed varied levels of regularity for both cutting speeds and all tool holders, which changed depending on the scale factor, making it difficult to clearly indicate the signal with the smallest level of disorder.

**Figure 21:**
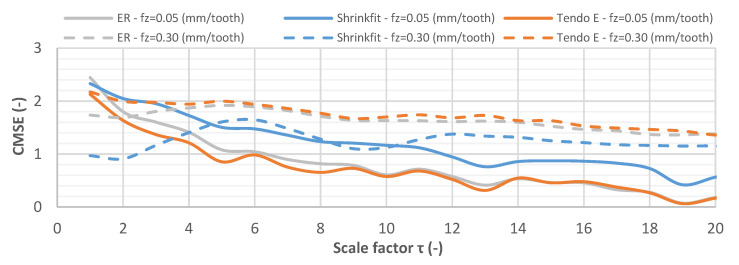
CMSE for vibration acceleration signals while milling at f_z_ = 0.05 mm/tooth and f_z_ = 0.30 mm/tooth (v_c_ = 800 m/min, a_p_ = 6 mm).

Figure 21 shows the CMSE curve for vibration acceleration signals while milling at a feed per tooth of f_z_ = 0.05 mm/tooth and f_z_ = 0.30 mm/tooth. The signals obtained for the respective tool holders and the feed per tooth values showed varied levels of regularity. The entropy value for the vibration acceleration signals in the course of milling at f_z_ = 0.05 mm/tooth decreased across the entire range of the scale factor, while for milling at f_z_ = 0.30 mm/tooth, an initial increase in entropy was observed, and its decrease occurred only at a scale factor of τ = 8–12.

## 4. Conclusions

Based on the research work and the analysis of the results, the following conclusions could be drawn:

- the assumed research aim of the study was achieved, it was possible to describe the dynamics of the milling of magnesium alloy with the use of basic vibrations indicators, as well as composite multiscale entropy (CMSE);
- the composite multiscale entropy (CMSE) method could be used to identify a complex dynamics of machining, particularly with respect to chatter phenomena;
- the selection of an appropriate configuration of technological parameters enabled a significant improvement in the stability of the magnesium alloy milling process;
- increasing the cutting speed and feed per tooth caused an increase in all values characterising the vibration displacement, except for v_c_ = 1000 m/min for which a decrease in the value was observed;
- the type of tool holder did not have a clear impact on the obtained values describing the vibration displacement;
- increasing the cutting speed and feed per tooth increased all the values describing the acceleration of vibrations;
- several times lower values characterising the vibration acceleration, by changing either the cutting speed or the feed per tooth, were obtained when the tool was mounted in the tool in a heat shrink holder;
- CMSE values for the vibration displacement signals increased by changing either the cutting speed or the feed per tooth, regardless of the tool holder used; however, the lowest entropy values occurred in the case of the heat shrink holder;
- CMSE values for the vibration acceleration signals decreased, achieving lower values for the signals recorded during the milling process, with the lowest feed per tooth value of f_z_ = 0.05 mm/tooth;
- the lowest values of vibration displacement x_y_ and their amplitude were observed at 400 m/min (0.016–0.018 mm), while similar values of x_y_ were observed at 1200 m/min (approximately 0.02 mm), which should be considered favourable from the point of view of machining efficiency;
- an increase in the cross-section of the undeformed chip, defined by an increase in f_z_, caused an increase in the value of x_y_ vibration displacement and amplitudes, and in the range of 0.05–0.2 mm/tooth, the range of x_y_ values covered the range of approximately 0.015–0.025 mm;
- the maximum values of vibration accelerations were the lowest at 400 m/min and were, respectively, for ER—16.9 m/s^2^, heat shrink chuck—4.9 m/s^2^, Tendo E—25.2 m/s^2^;
- when analysing the influence of f_z_, the vibration acceleration values increased approximately linearly, with the lowest a_y_ values obtained for 0.05 mm/tooth, and they were, respectively, for ER—23.7 m/s^2^, heat shrink chuck—5.7 m/s^2^, Tendo E—33.9 m/s^2^;
- the research carried out confirmed that magnesium alloys were characterised by a relatively high ability to damp vibrations, which ensured high stability of the machining processes performed, thus making it possible to increase their productivity.

## Figures and Tables

**Table 1:** The chemical composition of the AZ91D magnesium alloy.

Chemical Composition (wt.%)	Al	Zn	Mn	Si	Cu	Fe	Ni	Be	Mg
AZ91D	8.91	0.66	0.22	0.016	0.002	0.002	0.001	0.001	rest/others

## Data Availability

The raw/processed data required to reproduce these findings cannot be shared at this time as the data also form part of an ongoing study.

## Nomenclature

v_c_	cutting speed (m/min)
f_z_	feed per tooth (mm/tooth)
a_p_	axial depth of cut (mm)
a_e_	radial depth of cut (mm)
RMS	root mean square
CMSE	composite multiscale entropy
SLD	stability lobe diagram
x_y_/a_y_	maximum value of displacement, acceleration (mm or m/s^2^)
A_xy_/A_ay_	amplitude of displacement/acceleration (mm or m/s^2^)
x_y_RMS_/a_y_RMS_	root mean square value of displacement/acceleration (mm or m/s^2^)
λ_s_	helix angle (°)
m	the length of the chains
τ	scale factor
r	the similarity tolerance
σ_x_	the standard deviation of the original time series
